# Molecular profiling in contemporary breast cancer management

**DOI:** 10.1093/bjs/znad017

**Published:** 2023-02-08

**Authors:** Matthew G Davey, Michael J Kerin

**Affiliations:** Discipline of Surgery, The Lambe Institute for Translational Research, University of Galway, Galway, Ireland; Discipline of Surgery, The Lambe Institute for Translational Research, University of Galway, Galway, Ireland

Surgery remains the cornerstone of achieving breast cancer control, but understanding the biological and molecular processes that drive breast cancer development, coupled with the recent advances in therapeutic strategies, have facilitated an individualised approach to disease management. Even in the setting of hereditary breast cancer, risk-reducing surgery and novel targeted therapies have revolutionised management, with this being translated into enhanced oncological outcomes for the majority, including those with advanced disease. The rapidly evolving landscape of breast cancer management makes it imperative that breast surgeons’ knowledge of the biological disease spectrum aligns with contemporary practice. The aim of this review is to provide an update on how molecular cancer profiling influences the management of patients with primary breast cancer.

## Breast cancer molecular subtypes

Breast cancer was traditionally considered a homogenous disease entity that was best controlled with extensive surgical resection, before robust chemoendocrine and targeted radiotherapy were trialled and proven to enhance survival^1,2^. Molecular subclassification of tumours using the seminal 496-gene ‘intrinsic’ model highlighted biological differences and facilitated the practical division of breast cancers into clinically distinct subgroups, thus setting the foundations for the ‘molecular portraits of human breast cancer’^3^. Gene expression profiling initially subcategorised tumours broadly into four subgroups, namely ‘luminal-like’ cancers that were predominantly oestrogen and progesterone receptor positive (ER+/PgR+), human epidermal growth factor receptor-2 (or HER2/neu) positive, ‘basal-like’ (ER−/PgR−/HER2− that is triple-negative breast cancer (TNBC)), and ‘normal-like’ cancers^3^. Subsequent work from Sørlie *et al.* highlighted the pragmatism of subdividing the ‘luminal-like’ cancers into luminal A and B molecular subtypes, based on proliferation indices, treatment options, and expected prognoses^4^. This novel taxonomy recognized the heterogenous nature of breast cancer, with clinically and therapeutically distinct subtypes, leading expert consensus panels, and guidelines to endorse their adoption into routine clinical practice^5^. Where multigene signatures are unavailable^6^, immunohistochemical staining of core biopsies and tumour specimens act as surrogates for ER, PgR, HER2, and Ki-67/Mib-1 gene expression indices, and are used for individualized therapeutic decision-making during multidisciplinary discussion.

Neoadjuvant systemic therapy is increasingly recommended for both TNBC and HER2+ breast cancer, with several advantages, including tumour downstaging, increased eligibility for breast conservation surgery, and the generation of *in vivo* sensitivity data regarding the given systemic therapy. Currently, translational research efforts are focused upon the measurement of circulatory biomarkers, such as microRNAs, to further stratify breast cancer into novel molecular subtypes by predicting responses to neoadjuvant therapies^7,8^.

## Multigene expression assays

Contemporary breast cancer management uses multigene expression assays to personalise chemoendocrine prescription for those with early-stage ER+/HER2− disease. This practice refutes the hypothesis proposed by Bernie Fisher, a surgeon who believed breast cancer is a systemic disease that mandates treatment with systemic therapy^2^. The commercially available 21-gene and 70-gene assays are standardized and reproducible tests that perform a relative quantification of reverse transcriptase polymerase chain reaction products for key modulator genes in breast oncogenesis. These are then encompassed into an algorithm that gives as a result the likely to benefit from systemic chemotherapy^9,10^. Using the 21-gene expression assay, the seminal TAILORx and RxPONDER trials illustrated that the majority of postmenopausal patients with 0–3 positive lymph nodes gain no benefit from systemic chemotherapy^11,12^. This brings into question the necessity of performing routine sentinel lymph node biopsy to guide chemoendocrine prescription in such patients, once nodal disease has been reliably excluded clinically and/or radiologically. Similarly, results from the MINDACT trial demonstrate that patients considered to be high clinical and low genomic risk using the 70-gene assay share similar distant metastasis-free survival irrespective of chemotherapy use (95.9 per cent *versus* 94.4 per cent)^13^. These gene expression assays are endorsed by expert consensuses and recommendations, embedding their application into breast cancer management. Efforts to investigate the expansion of indications for these molecular signatures in novel settings are ongoing^14,15^, and include using such assays to predict tumour sensitivity to neoadjuvant therapies^16,17^.

## Genetic sequencing

In contemporary management, the most pertinent example illustrating the impact of genomic profiling on breast cancer surgery is the screening for pathogenic variants of the breast cancer gene 1 (*BRCA1*) and/or 2 (*BRCA2*). The risk of developing breast and ovarian cancers by the age of 70 is approximately 60 per cent and 59 per cent, respectively, for those with *BRCA1* alterations. The corresponding risks are 55 per cent and 17 per cent for those harbouring *BRCA2* alterations^18^. European Society for Medical Oncology and National Institute for Health and Care Excellence guidelines recommend that patients with known germline BRCA alterations should be counselled on the potential benefits of risk reduction surgery that includes prophylactic bilateral mastectomies and oophorectomies^19,20^, given the relative improvements in long-term overall (> 50 per cent) and breast cancer-specific survival (> 90 per cent) outcomes^21^. Moreover, genomic sequencing is now recommended by the National Collaborating Centre for Cancer in those considered to have a 10 per cent or greater a priori risk of *BRCA1* or *BRCA2* alterations, as shown by risk calculators (such as the Manchester criteria)^22^. Recently, the Breast Cancer Consortium highlighted the necessity to also treat variant in other genes, including among others *ATM*, *CHEK1*, and *CHEK2*, in a similar manner to BRCA alterations, given their important influence on breast cancer development^23^. Despite previous perceptions that primary breast cancer with BRCA alterations mandates mastectomy, there is now emerging evidence that breast-conservation surgery provides similar oncological outcomes^24^. Furthermore, current practice supports the use of poly-adenosine diphosphate-ribose polymerase (PARP) inhibitors, which target defects in homologous combination repair to enhance survival outcomes in both early- and advanced-stage BRCA-related breast cancers^25,26^.

## Future perspectives

Current practice focuses largely upon genomic and proteomic tumour analysis, but it is likely that future management strategies will see the establishment of therapeutically distinct biological subtypes that rely upon immunogenic and cellular regulatory enzymes as biomarkers/targets for further personalized care^27,28^. The advances described in this review encapsulate the impact the molecular era on personalising the management of primary breast cancer that has been translated into enhanced oncological outcomes.
